# The combined 410nm and infrared light effectively suppresses bacterial survival under realistic conditions

**DOI:** 10.3389/fcimb.2025.1624160

**Published:** 2025-08-01

**Authors:** Matthew Stangl, Dinesh Kumar Verma, Areli Martinez, Yong-Hwan Kim

**Affiliations:** ^1^ Department of Biological Sciences, Delaware State University, Dover, DE, United States; ^2^ Department of Biological Sciences, University of Delaware, Newark, DE, United States; ^3^ Neuroscience Program, School of Allied Health Sciences, Boise State University, Boise, ID, United States

**Keywords:** living space, 850nm, ROS induction, reduced biofilm, MDR bacteria, *E. coli*, and *S. aureus*

## Abstract

The demand for establishing an effective but inexpensive method to interfere with the spread of infectious diseases has been higher than ever before, since the recent pandemic. As a follow-up study, we tested a few practically applicable lights with a safe 410nm violet light (V) with infrared (IR, 850nm) under realistic conditions to identify an optimal light for suppressing pathogens. Our results indicate that 410nm violet light is as effective as the previously tested 405nm violet light with infrared (850nm). Therefore, we focused on optimizing combined lights (3V-1IR or 2.33V-1IR) with lower power level that is below 24 Watt. Using the Multi Drug Resistant (MDR) *Escherichia coli* (*E. coli*) and *Staphylococcus aureus* (*S. aureus*) from ATCC, we confirmed that the combined 20W light effectively suppressed the survival of both MDR bacterial strains on a smooth surface at the distance of 25cm, 50cm, 1m or 2m, which mimicked the realistic living spaces. As expected, the effectiveness was inversely proportional to the exposed distance. For example, the light exposure suppressed more than 91-97% of *E. coli* within 1–2 hours and 96-99% of *S. aureus* within 2–6 hours at short distances (25 or 50cm), whereas it took 6–8 hours to reach 92-95% of *E. coli* and 91-99% of *S. aureus* suppression at 1 or 2m. In the mechanistic studies, we confirmed that the bacterial death was mediated by the enhanced level of Reactive Oxygen Species (ROS), in addition to reduced thickness of biofilm from 410nm and 850nm infrared light. Our results strongly support the possible application of using this combined 410nm with infrared light as an inexpensive and practical solution to reduce the potential pathogens, at least from bacterial origins in a variety of living spaces.

## Graphical Abstract

Created by Dinesh K Verma in BioRender. Kim, YH. (2025) https://BioRender.com/e50i242

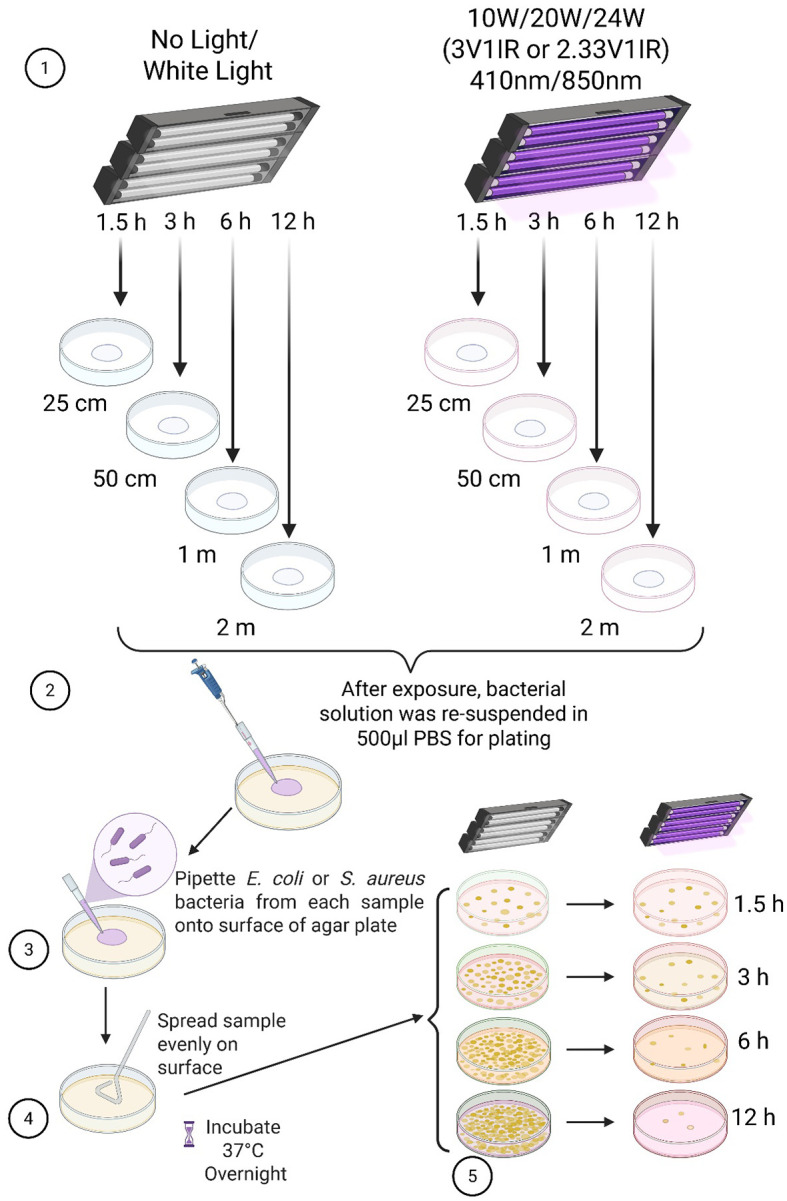

## Introduction

Even though the recent pandemic is over, COVID-19 driven global chaos left a lot of issues behind for us to deal with for many incoming years. A striking challenge we face is to develop creative methods to mitigate the potential risks for triggering high morbidity and mortality caused by bacteria- or virus-derived infectious diseases. Since dealing with infectious diseases can be risky, painful, costly and life-threatening, it is ideal to prevent the spread of infections via removing the potential pathogens before they become harmful (Indravudh et al., 2025; Walker et al., 2025). Although we have a few approaches, such as vaccines, antibiotics and medical treatments, to cope with a variety of infectious diseases, the best approach is to establish inexpensive set-ups to suppress potential pathogens in our living spaces (Fung et al., 2024; Choi et al., 2025). Lately, numerous research demonstrated that non-invasive applications of violet-blue lights prevent the potential infections or induce the healing process, which can be excellent solutions for public health (Jackson et al., 2024; Kruszewska-Naczk et al., 2024; Qin et al., 2024; Hur and Diez-Gonzalez, 2025; Tieman et al., 2025). In addition, the photothermal or photodynamic inactivation has been suggested for antimicrobial effects by applying near-infrared (780nm – 3000nm) light, which may show the additive effects of violet-blue lights (Dai et al., 2023; Tomás et al., 2025). Thus, our approach is focused on developing easy-to-use light combinations of violet and infrared to prevent the spread of potential pathogens (Ivanova et al., 2021; Serrage et al., 2024). Recently we reported the possibility of using safe and inexpensive light-emitting diode (LED) irradiation to reduce the risk of infection substantially due to the suppression of bacterial survival (Martinez et al., 2023). In the previous study, we have adopted a combined light between 405nm violet and 850nm infrared in the 3:1 ratio, which showed an effective suppression against multidrug-resistant (MDR)-Gram negative and positive bacteria. However, a concern was raised whether 405nm violet light is safe to apply broadly for humans to be exposed for extended period.

In this study, we are focused on developing even safer and more cost- & energy-efficient lights for practical applications in living spaces without having a potential risk. Since 405nm is relatively close to ultraviolet (UV) range (<400nm), and the previously applied lights were high-power lights (50 watt), which are not within the normal power level in our living spaces, such as office, home and hospital, *etc*., there is a need to modify the combined light with lower power and using a wavelength further away from the UV range. That was the primary motivation for us to develop and test new lights with lower than 24W and violet light that is further away from the UV range. Using the same MDR-bacteria (*Escherichia coli*: ATCC: BAA-2774 and *Staphylococcus aureus*: ATCC: BAA-1717) at the range of distance (25cm – 2m) within hours of exposure, we assessed a few safe lights combined with 410nm and 850nm in 3:1 or 2.33:1 ratio under 24W power level.

Our new approach is to use 410nm that is safer than 405nm with lower power levels (10–24 watts), instead of 50W. Previously, we demonstrated the reduced bacterial colonies after exposing the combined light (405 & 850nm) on agar plates, followed by incubation for counting the unit of colonies. However, it was questionable how closely applied experimental conditions would be relevant to the real living spaces we need to maintain nearly sterile. Therefore, the conditions we applied in this study were the exposure of new lights (410 & 850nm, 10-24W) at the distance of 25, 50cm, 1m and 2m for a short period of time (less than 6–8 hours) to test the feasibility of preventing bacterial contamination from the smooth surface in our real living spaces. In addition, we attempted to test if these combined lights are safe for mammalian cells *in vitro*. Furthermore, we assessed the underlying mechanisms of bacterial death by the combined lights through measuring the amount of Reactive Oxygen Species (ROS) generated and the reduced thickness of biofilms due to the violet (410nm) and infrared lights (Martinez et al., 2023). Our results strongly suggest that the combined lights in the ratio of 3:1 or 2.33 in V:IR LED lights effectively suppressed the survival of MDR-bacteria under realistic conditions, which support the broad application to prevent potential infectious diseases in various indoor spaces (Ivanova et al., 2021; Serrage et al., 2024).

## Materials and methods

### Light preparation

**Figure 1:**
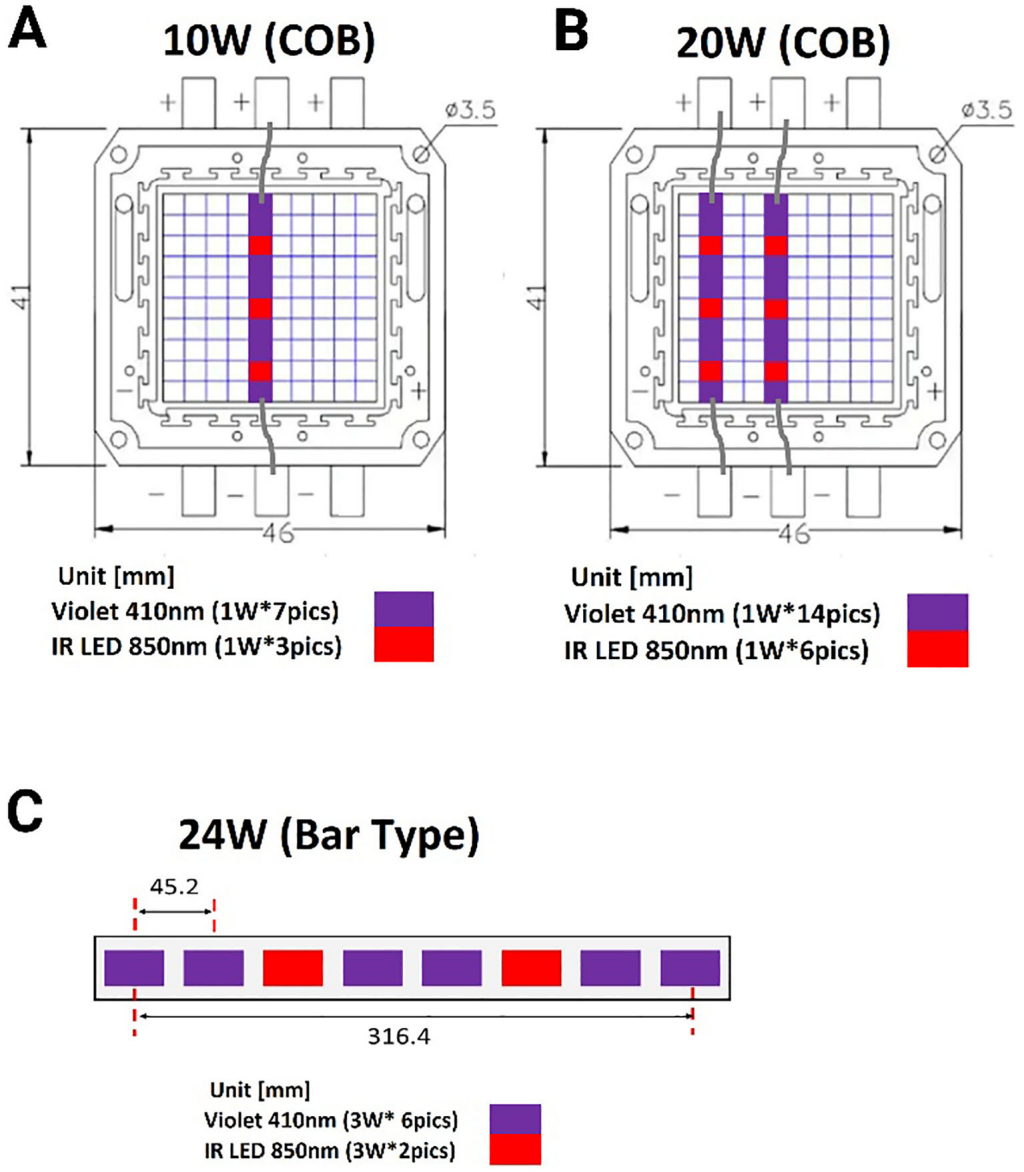
A schematic diagram of combined lights arranged in a different format based on power level. **(A)** The small LED lights (1W each) are arranged to constitute 10W in the ratio of 7:3 (410nm vs. 850nm) in a row. **(B)** Two rows of LED lights are parallelly lined up for 20W (14:6 = 410nm & 850nm). **(C)** A bar type of light is composed of eight 3W per light in the ratio of 3:1 of 410 vs. 850nm. COB: chip on the board.

Since our recent publication demonstrated that the 3:1 ratio light (3V-1IR) was effective in suppressing bacterial growth (Martinez et al., 2023), we decided to keep the ratio of 3:1 or less (2.33:1) between violet and infrared (850nm) in this follow-up study. However, due to the high-power level, 50W at 405nm in the previous lights, we requested the Analog Chip Production (ACP) Technology (San Ramon, CA) to redesign the lights below 24 Watt: 10, 20 or 24W and adopted 410nm to reduce the safety concerns and to improve the practical applicability in living spaces. All the LED lights were uniquely designed and developed by the ACP and applied for experiments. The amounts of power exposed at different distances are calculated and displayed in **Table 1**, and the layouts of lights are displayed in **Figure 1**.

**Table 1:** The exposed light intensities and areas for three different types of lights are calculated based on the distance and the illumination angle (60°).

Distance (meter)	Exposed Light Intensity (mW/cm^2^)			Exposed Area (m^2^)
	10W	20W	24W	
0.25	15.3	30.6	27.5	0.065
0.5	3.8	7.7	9.2	0.262
1	1	1.9	2.3	1.047
2	0.2	0.5	0.6	4.187

Radius (m) = tan_angle (60°/2) x distance (m), Exposed Area = 3.14 x radius (m)^2^, and Exposed Unit Power = W/area [W/m^2^] were calculated above as reported (Martinez et al., 2023).

## Reagents

In this study, most reagents, including Luria-Bertani Agar (LBA, BP1427-500, BP-160-500), Luria-Bertani Broth (LBB, MP-3002-132), Agarose (BP160-500), EZ rich medium, Tryptic Soy Broth (TSB, BD 211825), Brain Heart Infusion Broth (BHIB; Millipore 53286), Crystal Violet (C6158, Sigma-Aldrich) and Phosphate Buffered Saline (PBS, Gibco-20012-027) were used as reported (Parashar et al., 2013; Martinez et al., 2023) and purchased from Fisher scientific (Waltham, MA) for experiments.

### Light exposure to bacteria and colony counting

MDR-*E. coli* (ATCC: BAA-2774) is Gram-negative aerobic bacteria, and methicillin-resistant *S. aureus* (MRSA, ATCC: BAA-1717) is gram-positive aerobic bacteria. All the bacterial solutions from ATCC were prepared as we recently reported (Martinez et al., 2023). After measuring the density of bacteria at OD_600_, the log phase bacterial solution was diluted in the LBB to reach a consistent density: OD = ± 1.0, which contained 8 x 10^8^ CFU/mL of MDR-*E. coli* or 2 x 10^8^ CFU/mL of MRSA. To apply the consistent bacterial density, the concentrated bacterial solution was serially diluted in the sterile LB broth to create a master stock equal to 400 CFU/mL, after the bacteria were centrifuged and resuspended in an equal volume of PBS. A solution of MDR-*E. coli* or -*S. aureus* (250 µl) was dropped on a sterile empty petri-dish and exposed to three different 3:1 or 2.33:1 ratioed lights (10, 20 or 24W) at 4 different distances (25cm, 50cm, 1m or 2m) at room temperature (22-25°C) for up to 12 h. After light exposure was completed, partially dried bacterial solution was completely resuspended in 500 µl PBS and then smeared on LBA plates for incubation at 37°C overnight (Martinez et al., 2023). Each plate was seeded with fully resuspended bacterial solution in triplicates for counting the number of colonies in a blind manner (n=3/group in three independent experiments). Two control groups were included in the experiments, no light (in the dark) or 10W white light under the same conditions (Martinez et al., 2023).

### Measurement of ROS and biofilm thickness

As we previously reported (Martinez et al., 2023), the levels of ROS were measured using the CellRox deep red reagent (Invitrogen, C10422) (Yang and Choi, 2018). After picking a colony of MDR-*E. coli*, bacteria were grown in the EZ rich medium in a shaker at 37°C overnight. Then, *E. coli* was plated in 6 well plates containing EZ rich medium and exposed to 20W light with two controls (no light and 20W white light) for 1.5 h. After light exposure, 6 well plates were removed from the lights, and then CellRox deep red was added to each well and incubated for 30 min. For ROS measurements, the generated ROS levels by the lights were detected in fluorescence intensity, after excitation/emission at 644/665 nm, using the SpectraMax M5e plate reader (Molecular Devices). The intensity was normalized by the background level for comparison. Examples of ROS images of *E. coli* were captured using the EVOS FL (400x) system (Invitrogen) for comparisons and displayed with no light and white light controls.

For biofilm measurement, *E. coli* was grown in TSB overnight and back diluted to 0.025 at OD_600_ in BHIB supplemented with NaCl (4%, w/v). Two hundred μl of these cultures were added to sterile 96-well polystyrene plates (Fisher Scientific) and incubated at 37°C for 24 h. Wells were washed 3 times in PBS (pH 7.4) and dried by inversion at room temperature for 1 h (Parashar et al., 2013). Adherent bacteria were stained with 100 μl of 0.5% (w/v) crystal violet solution. After the stain was removed, the wells were washed 3 times in PBS (pH 7.4). Any adhering stain was solubilized with 100 μl of 5% (v/v) acetic acid before measuring the density at OD_620_. Three independent experiments were performed in triplicate, as reported (Parashar et al., 2013). The average and SEM of those three values from one representative experiment are depicted as reported (Sambanthamoorthy et al., 2012; Parashar et al., 2013).

### Mammalian cell culture

The N27 parental cell line was obtained from EMD Millipore (SCC048, Burlington, MA, USA), used only under 20 passage number and maintained in RPMI 1640 supplemented with 10% fetal bovine serum (FBS) and 1% Penicillin-Streptomycin at 37°C and 5% CO_2_ using standard cell culture methods (Verma et al., 2021). The total number of 0.5x10^6^ N27 cells were seeded the day before the experiment. Cells were exposed to 4V or 3V1IR light at 50cm in a CO2 incubator at 37°C ± 2 for 8, 16 and 32 hours (n=3). Cell viability was measured based on trypan blue staining using an automated cell counter Invitrogen Countess™ 3.

### Statistical analysis

**Figure 2:**
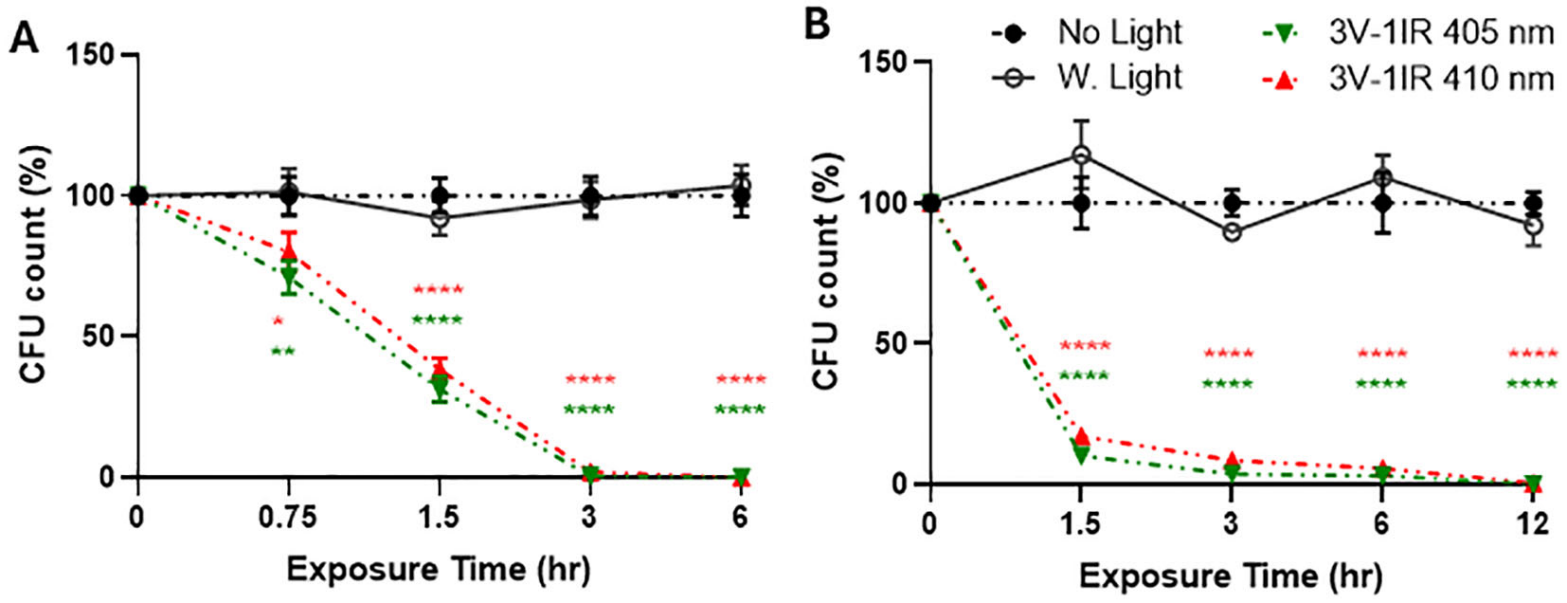
Both 405nm-IR and 410nm-IR effectively suppress the survival of *E. coli* on a smooth surface at 50cm **(A)** and 1m **(B)**. **(A)** 410nm-IR was nearly as effective as 405nm-IR to suppress the survival of *E. coli* at 50cm, after plating all the suspended bacterial solution cultured on LB broth. There is no significant difference in colony forming units (CFU) between 405nm and 410nm. **(B)** Both lights suppressed over 99.9% of *E. coli* survival by 12 h at 1m. Two-way ANOVA, Tukey’s HSD *post hoc* test was applied to show statistical significance, compared to the relativity of no light or white light (50W) exposure controls at each time-point (100% in relativity). *p<0.05, **p<0.01, ****p<0.0001 (* was displayed based on no light control using different colors representing each light, due to the tight space for clarity).

**Figure 3:**
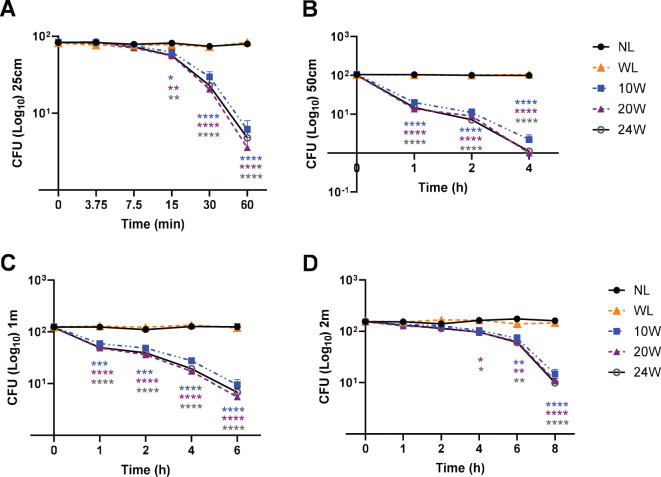
The combined 410nm with IR light exposure effectively terminates MDR-*E. coli* survival in a time-dependent manner at 25cm, 50cm, 1m and 2m. **(A)** At 25cm, over 96% of *E. coli* were terminated by 410nm-IR with 20W light, which was not significantly different from 10W or 24W. **(B)** At 50cm, over 99% of MDR-*E. coli* were suppressed by the 20W light within 4 h **(C)** At 1m, it took 6 h to terminate over 94% of *E. coli* with 20W light. **(D)** At 2m, the range of 91-93% of *E. coli* was eradicated by 20 or 24W light within 8 h Two-way ANOVA, Tukey’s HSD *post-hoc* test was applied to show statistical significance, compared to the relativity of no light exposure control. *p<0.05, **p<0.01, ***p<0.001, and ****p<0.0001 (* was displayed using different colors representing each light, due to the tight space for clarity).

**Figure 4:**
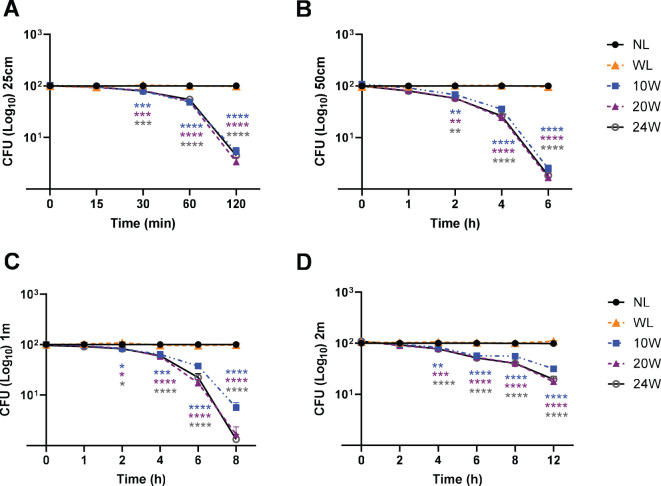
The 410nm-IR light terminates MDR-*S. aureus* survival in a time-dependent manner and inversely proportional to distance. **(A)** Within 2 h, 95-97% of *S. aureus* were suppressed by 20 or 24W light at 25cm. **(B)** At 50cm, around 99% of bacteria were suppressed by all three lights (10, 20 and 24W) within 6 h **(C)** At 1m, around 99% of *S. aureus* were suppressed by 20W and 24W lights within 8 h **(D)** At 2m, over 91.44% or 92.42% was terminated by 20W or 24W within 8 h, respectively. Two-way ANOVA, Tukey’s HSD *post-hoc* test was applied to show statistical significance, compared to the relativity of no light exposure control. *p<0.05, **p<0.01, ***p<0.001, and ****p<0.0001 (* was displayed using different colors representing each light, due to the tight space for clarity).

**Figure 5:**
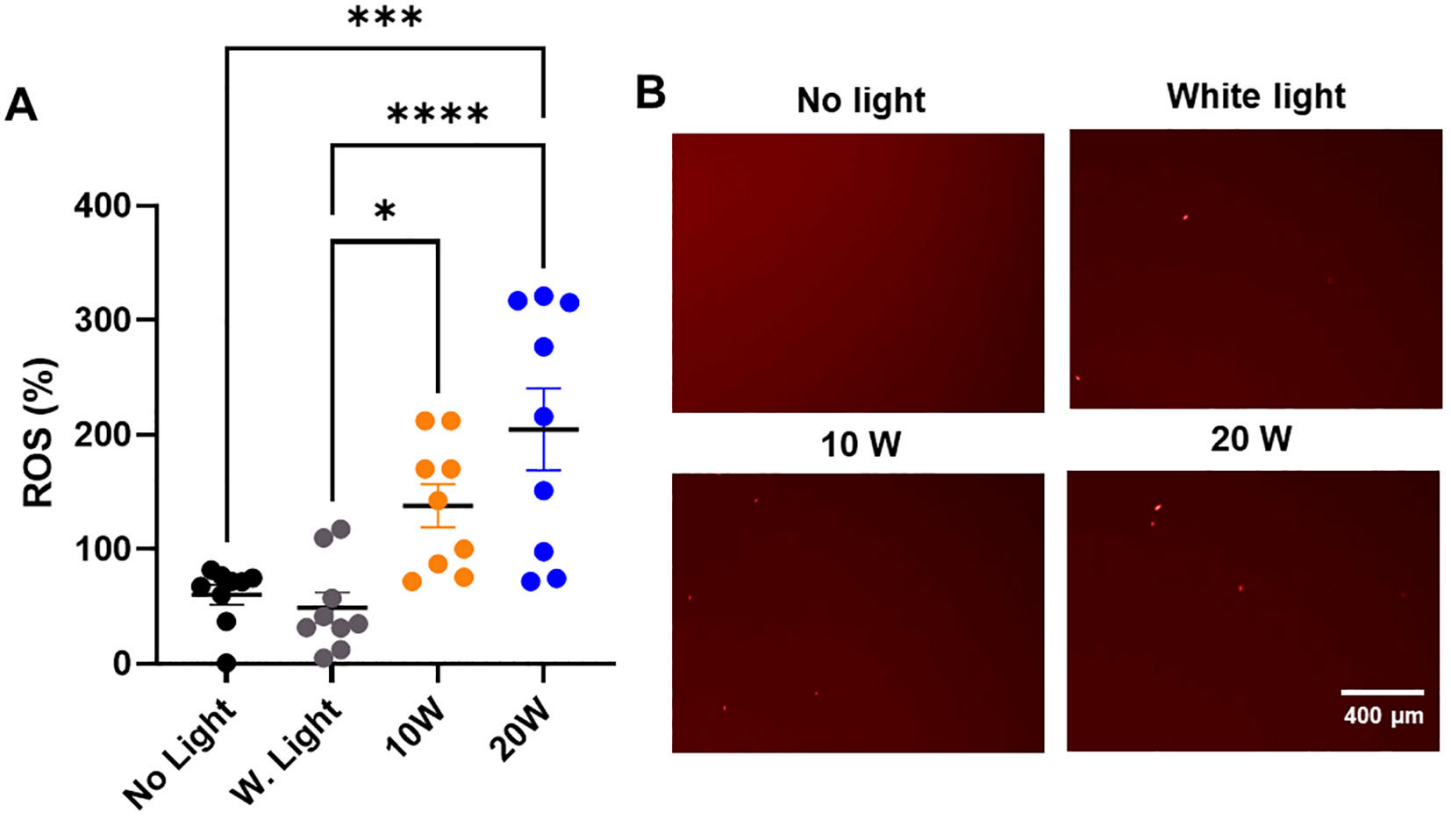
The 410nm-IR light induces the ROS generation in MDR-*E. coli* within 1.5 h at 50cm. **(A)** The 20W of combined light effectively induced the ROS generation at 50cm within 1.5 h, while 10W light was marginally efficient in generating ROS within 1.5 h (n=3x3). **(B)** The examples of ROS images in *E. coli* are displayed in different types of light exposure within 1.5 h at 50cm. One-way ANOVA, Tukey’s *post hoc* test was applied to show statistical significance, compared to the relativity of no light exposure control. *p<0.05, ***p<0.001, ****p<0.0001.

**Figure 6:**
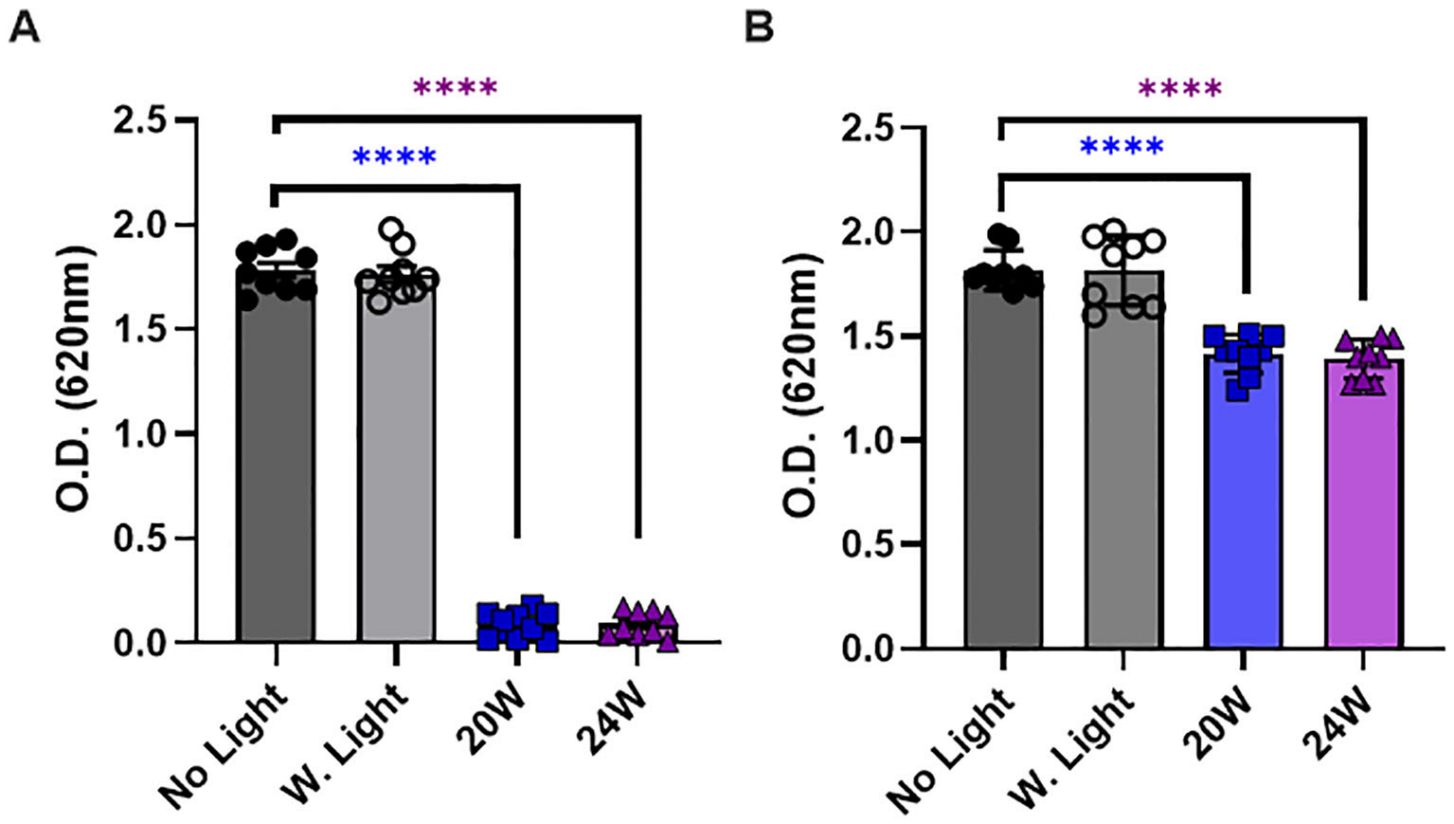
The 410nm-IR light reduces the thickness of biofilm in *E. coli* at 50cm within 1.5 hours. **(A)** The light exposure effectively prevented the biofilm formation in *E. coli* when bacteria were illuminated by the light before a colony formation. **(B)** The light exposure also partially induced the degradation of biofilm after bacterial colonies were formed. One-way ANOVA, Dunnett’s test was applied to show statistical significance, compared to the relativity of no light exposure control. ****p<0.0001.

The resuspended bacterial solution plated on LB plates was calculated by counting colonies by a blinded rater who is not familiar with light exposure conditions. The number of colonies from each light exposure was compared to no light or 10W white light control (n=3/group for 3 independent experiments) as a percentage of relativity (100%, #) in **Figure 2** or colony numbers in **Figures 3**, **4** at each time-point. Graph Pad Prism version 10.0 was used for statistical analysis. The presented results are displayed as the mean ± SEM using the analyses of two-way analysis of variance (ANOVA) with Tukey’s HSD pos-hoc test for multiple comparisons (**Figures 2**-**4**) or one-way ANOVA, with Tukey’s *post hoc* (**Figure 5**) or Dunnett’s test (**Figure 6**). The p-value below 0.05 was considered significant (*) throughout the entire study.

## Results

### The wavelength of 410nm is as effective as 405nm LED light to terminate the survival of *E. coli* at both 50cm and 1 meter

As a follow-up study (Martinez et al., 2023), we compared the effectiveness of 410nm combined with 850nm with that of previously tested 405nm with 850nm on 50W at 50cm (**Figure 2A**) and 1m (**Figure 2B**). Our assessment was based on two different negative controls, such as no light and white light (50W). In this study, three independent measurements were run in a triplicate to generate sufficient statistical powers (n=3x3). In **Figure 2A**, both 405 and 410nm with infrared effectively suppressed the growth of MDR-*E. coli* at 0.5m, compared to no light and white light-emitting diode (LED) controls, which were more effectively verified at 1m (**Figure 2B**). Due to the closer distance at 0.5m with higher power level, 405nm was slightly more effective than 410nm, however, the differences between 405nm and 410nm at 0.5m and 1m were subtle and not significant. At all the measured time points at 0.5 meter and 1 meter, both lights significantly suppressed the survival of MDR-*E. coli*, compared to no light and white light controls. For example, at 50cm, both lights terminated over 99% of *E. coli* within 3 h and over 99.99% by 6 h (**Figure 2A**), while at 1m, both lights showed over 94% suppression at 3 h, over 98% at 6 h, and over 99.9% by 12 h (**Figure 2B**).

### The light exposure of 2.33:1 or 3:1 ratioed 410nm with IR suppresses the survival of MDR-*E. coli* in a time-dependent manner and inversely proportional to distance

Since we verified that 410nm was nearly as effective as 405nm at 50W, our next assessment was to compare the effectiveness of low power lights (10, 20 and 24W) against MDR-*E. coli* from ATCC in the range of 25cm – 2m. At 25cm, our results verified that over 96% of *E. coli* were suppressed by 20W light (410nm-850nm in 2.33:1 ratio) (**Figure 3A**). There is no significant difference between 10, 20 and 24W, although subtle differences were observed in a power-level dependent manner. At 50cm, over 99.6% were suppressed by the 20W light within 4 h (**Figure 3B**). These results were similarly confirmed at 1m (**Figure 3C**) and 2m (**Figure 3D**). For example, over 94% of *E. coli* were suppressed within 6 h at 1m, and over 91% within 8 h at 2m, while no light and white light (20W) controls did not show significant changes in *E. coli* survival. The entire suppression rates are listed in the **Supplementary Table 1A** (25cm), 1B (50cm), 1C (1m) and 1D (2m).

### The 410nm-IR light exposure substantially eradicates MDR-*S. aureus* at various distances within 2–8 hours

In the following experiments, we have applied the same conditions against the Gram-positive MDR bacterial strain, *S. aureus* from ATCC. Using those 3 different lights (10, 20 or 24W), in addition to the 20W white light, we measured the effectiveness at 4 different distances. In **Figure 4A**, all the 410nm with IR lights (10, 20 and 24W) terminated over 95% of MDR-*S. aureus* survival within 2 h. At 50cm, 98-99% *S. aureus* were terminated by all the tested lights within 6 h, compared to no light or 20W white light (**Figure 4B**). At 1m, both 20W and 24W lights showed over 99% suppression within 8 h (**Figure 4C**). At 2m, both 20W and 24W lights effectively suppressed 91-93% of survival after 8 h of exposure, compared to no light or white light control (20W) (**Figure 4D**). The entire suppression rates of *S. aureus* are displayed in the **Supplementary Table 2A** (25cm), 2B (50cm), 2C (1m) and 2D (2m).

### The bacterial death is mediated by ROS generation from 410nm-IR light exposure within 1.5 hours

To understand the mechanism of bacterial death, we tested if 410nm-IR induced the ROS generation in *E. coli* to mediate bacterial death. In this experiment, we applied the same conditions as we used previously (Martinez et al., 2023). At 50cm, the same 410nm-IR light (20W) exposure for 1.5 h generated 3–4 folds (204.66 ± 107.59) higher levels of ROS than that of no light controls (60.19 ± 25.97) (**Figure 5A**). In the analysis, white light (20W) control (48.89 ± 39.71) was not different from no light control (**Figure 5A**). However, lower power 410nm-IR light (10W) was marginally elevated in ROS at 1.5 h post-exposure, which was enhanced significantly at 3 h post-exposure. In this assessment, we found that 20W 410nm-IR significantly enhanced the level of ROS after 1.5 h of light exposure. Examples of ROS staining images against MDR-*E. coli* are displayed in **Figure 5B**. As we reported previously (Martinez et al., 2023), 410nm violet light as well as 405nm induced ROS generation, which is at least a part of mechanism of inducing bacterial death by the light.

### The light exposure prevents the formation of biofilm and reduces the thickness of formed biofilm in *E. coli*, which contributes to bacterial death

In our last experiments, we assessed whether the light exposure reduces the thickness of biofilm in *E. coli* or not. Our assessment is intended to distinguish whether the light exposure prevents the biofilm formation in a proliferative stage, or it reduces the thickness of biofilm even after a colony and biofilm were fully formed at 50cm. Thus, our experimental design was to expose the light right after plating bacteria on a plate for assessing the preventive effects or to expose the light to *E. coli* after a colony was visibly formed. Since we detected a marginally significant increase in ROS generation by 10W for 1.5 h (**Figure 5A**), we excluded the 10W light from the test and measured the responses in biofilm by 20W and 24W, which were compared with no light (dark gray) and white light (20W, light gray) controls. In **Figure 6A**, we show that both 20W and 24W significantly prevented the biofilm formation in MDR-*E. coli* after 1.5 h of light exposure at 50cm. In **Figure 6B**, the light exposure was applied to fully formed *E. coli* colonies for 1.5 h at the same distance (50cm). The thickness of biofilm was measured after labeling biofilm specifically and followed by density measurement at OD_620_ (Sambanthamoorthy et al., 2012; Parashar et al., 2013). Our results indicated that the light exposure significantly prevented the formation of biofilm (**Figure 6A**), while the formed biofilm was also partially degraded by the light exposure, although the magnitude was marginally significant (**Figure 6B**).

## Discussion

In our previous study, we investigated the potential application of violet-blue light between 405 and 450nm for anti-bacterial effects. Our findings support the effectiveness of 405nm over 450nm, thus we adopted 405nm for violet light in the previous study (Martinez et al., 2023). According to the published studies, we expected that 405nm is more effective than 410nm in suppressing bacterial growth or survival (Tran et al., 2018; Sinclair et al., 2023, 2024). Thus, our initial assessment in this study was to compare the effectiveness of preventing bacterial growth between 405nm and 410nm (**Figure 2**). Interestingly, the difference was nearly negligible in our results against *E. coli* and *S. aureus*. Therefore, we decided to adopt 410nm, instead of using 405nm because 410nm would be safer than 405nm, due to further away from the UV range (Katayama et al., 2018; Morimoto et al., 2014; Martegani et al., 2020). Our next concern was that applying 50W light indoors would be beyond the comfortable range of brightness and generating heat in the exposed regions, in addition to high cost. Thus, we decided to test the effectiveness of combined lights at lower power levels (lower than 24W). In our current assessments, we used 10-24W lights that are 2.33:1 or 3:1 ratio of 410nm:850nm in combined lights. The other concern we have is whether these combined lights are safe for humans or not, especially after a long-term exposure in a short distance. Thus, we exposed the light to mammalian cells, N27 rat dopaminergic cells for 8, 16 and 32 hours, to assess if cell growth would be interrupted by the light exposure. Even the 50W high power lights at the distance of 50cm we used for the previous report (4V or 3V1IR) did not interfere with the normal growth of N27 cells, compared to white light control (**Supplementary Figure 1**). Although our negative results may not assure the safeness of the combined lights, and it remains to be tested using *in vivo* models, it appears to be optimistic to use the light in our living spaces.

In this study, we confirmed that the difference in anti-bacterial effects between 405 nm and 410nm was negligible under the circumstances (Imada et al., 2014; Halstead et al., 2016; Stewart et al., 2022). Against Gram-negative (*E. coli*) and -positive MDR-bacteria (*Staphylococcus aureus*), the energy efficient LED light effectively suppressed (>99%) the bacterial survival at 1m within 6 hours for *E. coli* and within 8 hours for *S. aureus*. As expected, its effectiveness is inversely proportional to the exposure distance due to the power level (Martinez et al., 2023), however, even at 2m distance, its effect (>91%) against *E. coli* was clearly detected within 8 hours and over 81% against *S. aureus* within 12 hours. Therefore, we validated the potential applications of the combined lights to suppress bacterial or even microbial growth in our various living spaces. Since this light effectively terminated bacterial survival at 1m or shorter distance within reasonable hours (<6h), this light is optimal for short distance (up to 1m). Thereafter, there is a need to redesign the light to further optimize at a short distance, which encourages additional modifications for improving the effectiveness.

Although the mechanism of bacterial death by violet-blue lights are well studied (Lubart et al., 2011; Aponiene and Luksiene, 2015; Yang and Choi, 2018), we verified that the combined light sufficiently induced the ROS generation inside of bacteria for the photo-excitation effect by 410nm (Wang et al., 2019; Martinez et al., 2023). According to several published studies, the violet light stimulates a photosensitizer, porphyrins that are located in cytosol, or bacterial membrane- or cytoplasmic membrane-bound proteins, depending on the bacterial species and the type of porphyrins, resulting in oxidative stress in cytosol or inside of bacteria for damage (Kleinpenning et al., 2010; Aponiene and Luksiene, 2015; Machado et al., 2022; Xia et al., 2022; Qin et al., 2024). This photodynamic inactivation of bacteria is correlated with the elevated level of ROS for bacterial death (Imada et al., 2014; Maclean et al., 2014; Biener et al., 2017; Stewart et al., 2022; Qin et al., 2024). The antimicrobial effect is likely derived from oxygen-dependent photoexcitation by porphyrin activation and release into cytosol (Lubart et al., 2011; Kim et al., 2013; Maclean et al., 2014; Aponiene and Luksiene, 2015). This non-selective photodynamic inactivation targets most microbial organisms for suppressing their survival, which is an extremely effective and cost-efficient tool to prevent infectious diseases (Maclean et al., 2014; Stewart et al., 2022). Additionally, the mild heat and near infrared wavelength from infrared can contribute to dehydration of microorganism and additional antimicrobial effects, by which bacterial death would be accelerated, in addition to reducing unpleasant odor from bacterial by-products (Chiemchaisri et al., 2007; Mamone et al., 2024; Xuan et al., 2025). Furthermore, the combined light prevented biofilm formation, if the light is exposed to the bacteria before colonies were formed, whereas the thickness of biofilm was significantly reduced, even after fully formed colonies were exposed to light. As we reported recently (Martinez et al., 2023), the combined light (3:1 or 2.33:1 ratio in 410:850nm) triggered ROS generation and reduced the thickness of biofilm in bacteria for termination. Our results strongly support that the combined light of 410nm with 850nm effectively generated ROS in bacteria and prevented biofilm generation and further reduced the formed biofilm in *E. coli* (Li et al., 2019; Sun et al., 2022; Qin et al., 2024).

In summary, our results indicate that the light exposure of the safe violet (410 nm) light and infra-red (850 nm) in 2.33 or 3:1 ratio effectively suppressed the survival of both MDR-*E. coli* and -*S. aureus* under the realistic living conditions. The potential mechanism of bacterial death is mediated by ROS generation inside of bacteria, in addition to reduce the thickness of biofilm. Although the light exposure reduced the thickness of biofilm in fully grown *E. coli*, the preventive effects on biofilm formation effectively terminated the survival of *E. coli*, which contributed to the higher vulnerability for bacterial survival. Our results strongly support the idea of introducing easy-to-use and adaptable applications of safe violet light with IR, which would have a broad impact on preventing biofilm generation and inducing ROS generation in microorganisms to suppress bacterial survival. The potential applications of 410nm with 850nm in the range of using 20W lights would be feasible for a variety of living/working spaces, such as hospital, office, kitchen and other indoor spaces under realistic conditions, to substantially reduce a variety of infectious diseases.

## Author’s note

This study was performed in collaboration between the Kim lab at Delaware State University, currently at Boise State University and the Analog Chip Production (ACP) Technology. Although this study was framed by Y-HK and the ACP for the collaborative project, ACP has no relation to data collection, analyses and interpretation, and the manuscript writing for publication.

## Data Availability

The original contributions presented in the study are included in the article/**Supplementary Material**, further inquiries can be directed to the corresponding author/s.
