# Supplementary material for: The combined 410nm and infrared light effectively suppresses bacterial survival under realistic conditions
Source: Front Cell Infect Microbiol. 2025 Aug 1;15:1624160. doi: 10.3389/fcimb.2025.1624160 (PMC12355657; doi:10.3389/fcimb.2025.1624160)
Supplement: Supplementary file 1 [file DataSheet1.pdf]

Supplementary Materials

Note: In the supplementary tables 1-8, the Mean ± SEM are displayed in the percentage of dead bacterial colonies (CFU). The initial time points, 0 minute (min) or hour (h) are not shown because all bacterial colonies are considered intact without having dead bacteria, indicating "0" consistently.

1 Supplementary Tables 1A: The termination rates of *E. coli* are listed in a time-dependent manner at 25cm. (Mean ± SEM)

Time (min)	10W	20W	24W	White light
**3.75**	1.68±13.77	4.71±8.35	-0.75±11.98	6.65±16.20
**7.5**	4.29±11.66	10.40±7.64	8.11±10.68	3.05±17.00
**15**	23.37±12.45	31.15±10.99	30.92±15.88	5.05±12.54
**30**	61.17±15.25	73.21±11.08	69.95±13.61	1.73±17.60
**60**	93.19±5.98	96.63±4.25	95.31±6.26	-5.87±30.32

2 Supplementary Tables 1B: The termination rates of *E. coli* are listed in a time-dependent manner at 50cm. (Mean ± SEM)

Time (h)	10W	20W	24W	White light
**1**	81.26±11.65	86.54±6.99	85.34±8.81	0.89±16.84
**2**	88.66±8.27	90.85±7.56	92.62±6.70	-2.08±29.83
**4**	97.93±2.59	99.66±0.51	99.43±0.76	-5.89±20.91

3 Supplementary Tables 1C: The termination rates of *E. coli* are listed in a time-dependent manner at 1m. (Mean ± SEM)

Time (h)	10W	20W	24W	White light
**1**	52.73±20.08	59.21±12.12	58.83±9.49	-2.57±27.78
**2**	48.05±8.69	65.53±12.59	62.14±14.16	-11.34±25.46
**4**	72.59±8.02	84.48±7.82	82.93±9.12	-5.91±29.96
**6**	89.29±7.03	94.39±3.98	93.29±5.37	1.09±22.68

4 **Supplementary Tables 1D: The termination rates of *E. coli* are listed in a time-dependent manner at 2m.** (Mean ± SEM)

Time (h)	10W	20W	24W	White light
**1**	8.61±22.73	17.58±9.75	15.23±15.41	3.73±20.81
**2**	7.39±23.61	15.01±17.93	17.84±20.32	-17.65±35.84
**4**	34.73±15.48	40.82±12.50	40.75±11.15	-3.79±24.44
**6**	56.19±16.61	62.38±15.34	64.09±14.29	15.79±19.16
**8**	88.09±11.94	91.44±9.77	92.41±7.89	8.46±16.05

5 **Supplementary Tables 2A: The termination rates of *S. aureus* are listed in a time-dependent manner at 25cm.** (Mean ± SEM)

Time (min)	10W	20W	24W	White light
**15**	7.78±14.02	7.44±4.67	4.33±6.14	5.22±8.41
**30**	21±6.86	17.44±7.18	20.89±5.67	-3.78±13.25
**60**	51.67±6.93	50.44±5.77	45.67±3.74	0.56±8.88

**120**	94.78±3.42	96.89±2.67	95.67±3.87	2.11±6.35

6 **Supplementary Tables 2B: The termination rates of *S. aureus* are listed in a time-dependent manner at 50cm.** (Mean ± SEM)

Time (h)	10W	20W	24W	White light
**1**	15.42±21.15	19.31±11.21	19.93±13.92	-1.29±17.82
**2**	32.49±5.79	41.13±6.71	42.76±8.77	-0.16±8.14
**4**	65.75±6.33	75.71±6.43	70.08±13.72	-4.29±12.49
**6**	98.35±1.51	99.02±1.12	98.79±1.10	3.13±13.10

7 **Supplementary Tables 2C: The termination rates of *S. aureus* are listed in a time-dependent manner at 1m.** (Mean ± SEM)

Time (h)	10W	20W	24W	White light
**1**	4.96±16.67	4.91±11.23	5.42±13.23	-2.49±22.51
**2**	14.43±11.87	18.78±7.86	19.14±9.90	-2.83±13.44
**4**	32.37±11.57	40.71±8.51	38.56±10.50	3.63±11.22
**6**	67.41±10.18	81.49±6.30	78.03±8.50	0.36±15.25
**8**	94.25±3.71	98.99±1.10	99.33±0.82	0.84±20.80

8 **Supplementary Tables 2D: The termination rates of *S. aureus* are listed in a time-dependent manner at 2m.** (Mean ± SEM)

Time (h)	10W	20W	24W	White light
**1**	8.61±22.73	17.58±9.75	15.23±15.41	3.73±20.81

**2**	7.39±23.61	15.01±17.93	17.84±20.32	-17.65±35.84
**4**	34.73±15.48	40.82±12.50	40.75±11.15	-3.79±24.44
**6**	56.19±16.61	62.38±15.34	64.09±14.29	15.79±19.16
**8**	88.09±11.94	91.44±9.77	92.41±7.89	8.46±16.05

9 The relative levels of ROS were measured after 1.5 hours of light exposure.

Time (h)	No Light	W. Light	10 W	20 W
1.5	60.19±25.97	48.89±39.71	137.95±56.37	204.66±107.59

Supplementary Figure 1

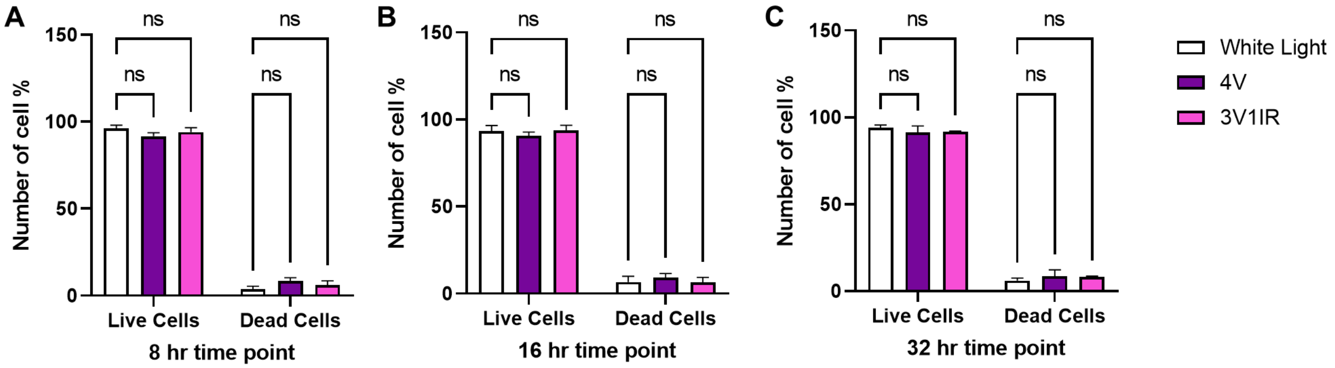

SUPPLEMENTARY FIGURE 1. The 4V or 3V1IR did not interfere with the normal growth of N27 rat dopamine cells, compared to white light control. Data are presented as mean ±SEM and analyzed by using GraphPad Prism 10.01 software using One ANOVA, Dunnett's multiple comparisons test.
